# Mechanistic insights into the role of amyloid-β in innate immunity

**DOI:** 10.1038/s41598-024-55423-9

**Published:** 2024-03-05

**Authors:** Tatum Prosswimmer, Anthony Heng, Valerie Daggett

**Affiliations:** 1 Molecular Engineering Program, University of Washington, Seattle, WA 98195-5610 USA; 2 Department of Neuroscience, University of Washington, Seattle, WA 98195-5610 USA; 3 Department of Biochemistry, University of Washington, Seattle, WA 98195-5610 USA; 4 Department of Bioengineering, University of Washington, Seattle, WA 98195-5610 USA

**Keywords:** Alzheimer’s disease, Amyloid, Biofilm, α-sheet, Neuroinflammation, β-Amyloid, Oligomer, Microbe, Alzheimer's disease, Bacterial host response

## Abstract

Colocalization of microbial pathogens and the β-amyloid peptide (Aβ) in the brain of Alzheimer’s disease (AD) patients suggests that microbial infection may play a role in sporadic AD. Aβ exhibits antimicrobial activity against numerous pathogens, supporting a potential role for Aβ in the innate immune response. While mammalian amyloid is associated with disease, many bacteria form amyloid fibrils to fortify the biofilm that protects the cells from the surrounding environment. In the microbial AD hypothesis, Aβ aggregates in response to infection to combat the pathogen. We hypothesize that this occurs through toxic Aβ oligomers that contain α-sheet structure and form prior to fibrillization. De novo designed α-sheet peptides specifically bind to the α-sheet structure present in the oligomers of both bacterial and mammalian amyloidogenic proteins to neutralize toxicity and inhibit aggregation. Here, we measure the effect of *E. coli* on Aβ, including upregulation, aggregation, and toxicity. Additionally, we determined the effect of Aβ structure on *E. coli* amyloid fibrils, or curli comprised of the CsgA protein, and biofilm formation. We found that curli formation by *E. coli* increased Aβ oligomer production, and Aβ oligomers inhibited curli biogenesis and reduced biofilm cell density. Further, curli and biofilm inhibition by Aβ oligomers increased *E. coli* susceptibility to gentamicin. Toxic oligomers of Aβ and CsgA interact via α-sheet interactions, neutralizing their toxicity. These results suggest that exposure to toxic oligomers formed by microbial pathogens triggers Aβ oligomer upregulation and aggregation to combat infection via selective interactions between α-sheet oligomers to neutralize toxicity of both species with subsequent inhibition of fibrillization.

## Introduction

**Figure 1:**
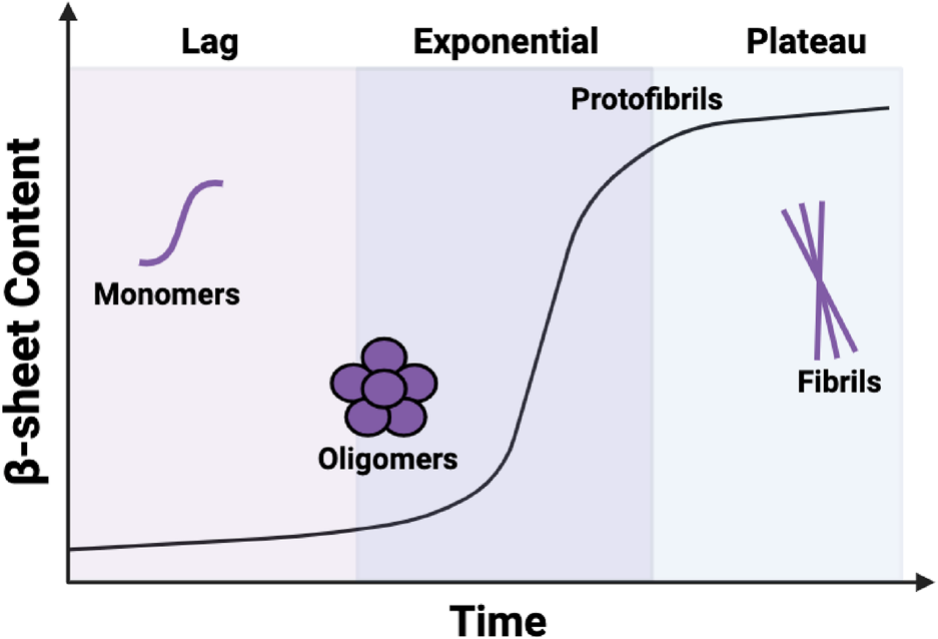
Amyloidogenesis of both Aβ and CsgA begins from a random coil, intrinsically disordered state and the formation of an aggregation-competent α-sheet monomer in the lag phase. At the end of the lag phase, the amyloidogenic protein changes structure from low molecular weight α-sheet soluble oligomers to β-sheet protofibrils. The plateau phase describes the stage at which fibrils have rearranged to adopt highly ordered cross-β-sheet structure. While random coil, α-sheet, and β-sheet conformers are enriched in the aggregation stages described above, primarily α-sheet mixed with random coil in the lag phase until the α-sheet becomes dominant just prior to the exponential phase. Similarly, some α-sheet remains in the beginning of the plateau, mixed with protofibrils. See Shea et al.^3^ for a breakdown of the structural transitions and further discussion.

Alzheimer’s disease (AD) is the sixth leading cause of death in the United States and is characterized by neuronal death and progressive loss of cognitive function^1,2^. Disease progression is associated with the aggregation of the 42-amino acid amyloid-β peptide (hereafter referred to as Aβ) (Fig. 1), which is intrinsically disordered in its ‘normal’ biologically active monomeric state^3^. During amyloidogenesis, Aβ forms toxic, soluble oligomers—implicated in neuronal death and disease pathology—prior to the deposition of mature fibrils^3–7^. Previously, Aβ aggregation was considered an inherently aberrant process, but recent studies suggest that aggregation may be triggered as a protective mechanism against microbial infection in the brain^8–18^.

Neuroinflammation has long been associated with AD and other diseases including Parkinson’s disease (PD), amyotrophic lateral sclerosis (ALS), and multiple sclerosis (MS), suggesting a central role of a sustained inflammatory response in neurodegenerative disorders^10,19–30^. Chronic inflammation in AD is attributed to a disruption in the balance of anti-inflammatory and pro-inflammatory signaling, resulting in chronic microglial cell activation and increased cytokine release^10,30–34^. Aβ aggregates are regularly degraded and phagocytosed by microglia, but when Aβ levels are significantly elevated, as is the case with AD, chronic activation of microglia results in a sustained pro-inflammatory response that exacerbates AD pathology and neuronal death^10,30,35,36^. The neuroinflammatory response then reduces Aβ degradation and phagocytosis by microglia, further elevates microglial activation, and produces a cyclical loop of neurodegeneration^10,30^. The mechanism that triggers the initial upregulation of Aβ aggregation is still largely uncharacterized, but evidence of microbial pathogens in AD patient brain samples suggests that Aβ aggregation is employed as an innate immune response to microbial infection^8,10,12,16–18,26^. The microbial AD hypothesis postulates that although Aβ aggregation is likely a programmed immune response to microbial pathogens, the excess buildup of toxic Aβ aggregates results in a chronic inflammatory response that causes AD pathology^8–10^. Notably, research suggesting that microbial infection triggers various types of dementia is well documented. Enterovirus has been shown to cause rapidly progressive dementia (RPD)^37^, while neuroborreliosis frequently leads to secondary dementia^38^. Other infections such as syphilis and cysticercosis have been implicated in cases of “reversible dementia” that abate with proper treatment such as intravenous antibiotic treatment^39,40^.

The microbial AD hypothesis was proposed based on numerous studies observing colocalization between microbial pathogens and Aβ aggregates in various brain regions of AD patients, including herpes simplex virus 1 (HSV1) DNA, bacterial lipopolysaccharide (LPS), and *Porphyromonas gingivalis*^16–18^. Additional pathogens that have been found in postmortem AD brains include archaea, chloroplastida, and holozoa^41^. Similar colocalization of Aβ and the previously mentioned pathogens was not observed in age matched control samples^16–18^. Additionally, Aβ exerts antimicrobial activity against numerous pathogens including *Escherichia coli* (*E. coli*), *Candida albicans (C. albicans)*, *Staphylococcus aureus (S. aureus)*, *Pseudomonas aeruginosa (P. aeruginosa)*^12,13^.

Aβ is believed to act as an antimicrobial peptide by entrapping pathogens such as bacteria, fungi, and viruses in extracellular fibrillar proteins, thereby protecting the host cells from the invading infection^8,11^. In one study, HSV1 seeded Aβ fibrillization in transgenic mice that produce human Aβ (5XFAD) and a 3D human neural cell culture infection model, and Aβ entrapped the pathogen inside plaques to protect surrounding neurons from the infection^11^. We hypothesize that the oligomeric form of Aβ functions as the first defense against pathogens (as they are the primary toxic species) prior to ‘entrapment’ by fibrils^3–7^. Further, we hypothesize that in the case of bacteria, Aβ oligomers combat pathogens by inhibiting their amyloid formation, therefore weakening the infection, and shifting cells from the protective biofilm to the planktonic, or free-floating, phase. In this hypothesis, bacterial amyloid inhibition by Aβ oligomers renders the bacteria more susceptible to the host immune response and subsequent entrapment by nontoxic Aβ fibrils and plaques.

Soluble oligomers of various amyloid species, including Aβ, CsgA (formed by *E. coli*), PSMα1 (formed by *S. aureus*), and more, have been shown to adopt a nonstandard secondary structure known as α-sheet^3,42,43^. Further, de novo designed α-sheet peptides inhibit amyloid formation and oligomeric toxicity by selectively binding to aggregates with the same structure, notably the toxic oligomers^3,42–46^. Bacterial amyloid inhibition by these de novo designed α-sheet peptides has also been shown to reduce biofilm cell density in *E. coli* and *S. aureus*, rendering the bacteria more susceptible to various antibiotics^43^.

**Figure 2:**
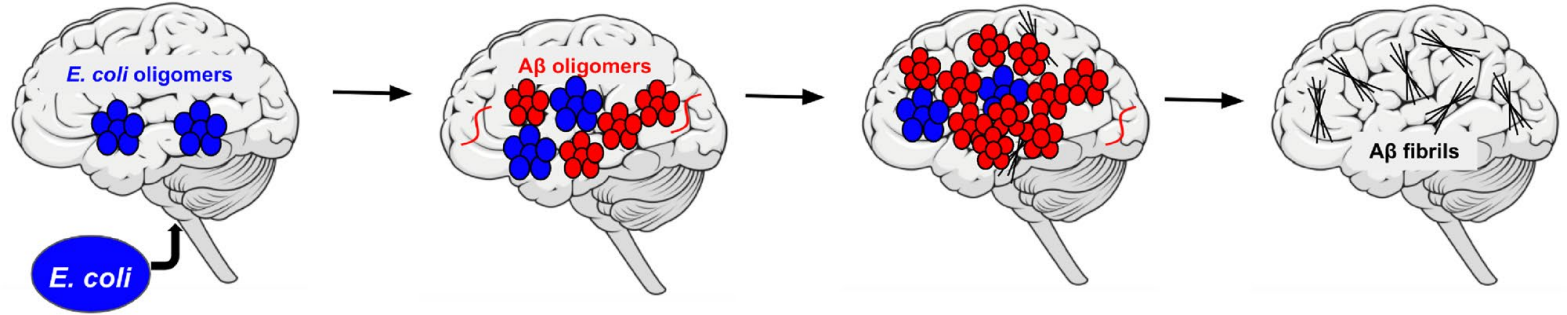
α-Sheet-mediated microbial Alzheimer’s disease hypothesis. *E. coli* crosses the blood brain barrier and deposits in brain tissue. The bacteria begin to form a surface-associated biofilm and secrete toxic, α-sheet containing oligomers (blue) in the process of forming amyloid. Exposure to these toxic oligomers elicits Aβ oligomerization (red) to inhibit curli formation through α-sheet interactions. Overaccumulation of toxic, α-sheet containing Aβ oligomers causes cell death. Finally, Aβ forms mature fibrils (gray).

Previous studies suggest that bacterial and mammalian amyloid proteins can inhibit one another’s aggregation, and inhibition is likely mediated by oligomeric species^47^. Transthyretin (TTR), a mammalian protein involved in various amyloid diseases including familial amyloidotic cardiomyopathy^47–49^, inhibits CsgA fibrillization by sequestering the bacterial protein into ‘dead-end’ oligomers^47^. Monomeric TTR (M-TTR), which rapidly oligomerizes into α-sheet aggregates^45,46,49^, was also shown to significantly inhibit uropathogenic *E. coli* biofilm formation^47^. Here, we investigated the effect of amyloid formation by uropathogenic *E. coli* on Aβ aggregation in neuroblastoma cells using the soluble oligomer binding assay (SOBA). SOBA is an ELISA-like assay that uses a de novo designed α-sheet peptide as the capture agent, thereby exhibiting specificity for low-molecular weight α-sheet soluble oligomers without binding to monomeric, random coil Aβ or fibrillar, β-sheet Aβ^50^. As such the assay provides a readout for the presence of α-sheet toxic oligomers^3^. We also performed biofilm assays with Thioflavin T (ThT), a fluorescent dye that binds β-sheet fibrils^51^, using a previously developed biofilm ThT protocol^42,43^ to measure the capacity for Aβ oligomers to inhibit *E. coli* biofilm and fibril formation. The α-sheet-mediated microbial AD hypothesis is described in Fig. 2.

## Results

### Amyloid-forming *E. coli* upregulate formation of Aβ α-sheet oligomers

**Figure 3:**
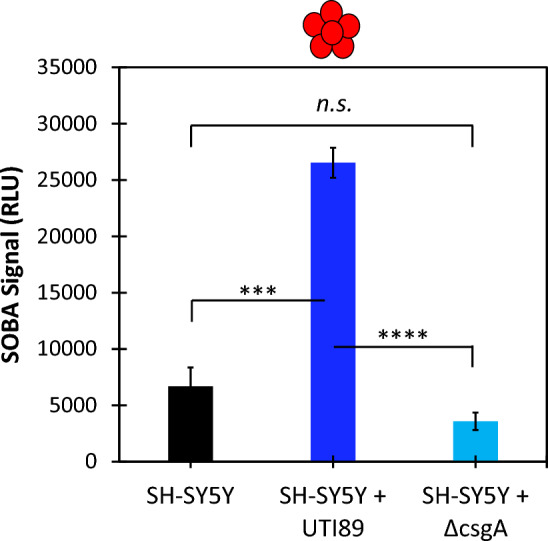
UTI89 upregulates α-sheet containing Aβ oligomers in neuroblastoma cells. Incubation with UTI89 resulted in a threefold increase (*p* = 0.0001) in the production of α-sheet-containing Aβ oligomers by neuroblastoma cells as compared to the control condition (SH-SY5Y neuroblastoma cells with media instead of bacteria). Incubation with ∆csgA caused a 50% decrease in the production of Aβ oligomers, although the result is not statistically significant (*p* *=* 0.07).

Neuroblastoma cells were grown with two strains of uropathogenic *E. coli*: UTI89, a robust amyloid- and biofilm-forming clinical isolate, and UTI89 ∆csgA, an engineered deletion strain that lacks the CsgA protein required to form curli fibrils (referred to below as ∆csgA). Following *E. coli* biofilm maturation, the planktonic phase was removed and evaluated with SOBA to determine whether toxic α-sheet Aβ oligomers were present in solution. Incubation with amyloid-forming *E. coli* (UTI89) led to a threefold increase in the formation of Aβ oligomers. The SOBA signal decreased approximately 50% when neuroblastoma cells were grown with UTI89 ∆csgA, although the result is not statistically significant (Fig. 3).

### Aβ oligomers inhibit curli formation by *E. coli*

**Figure 4:**
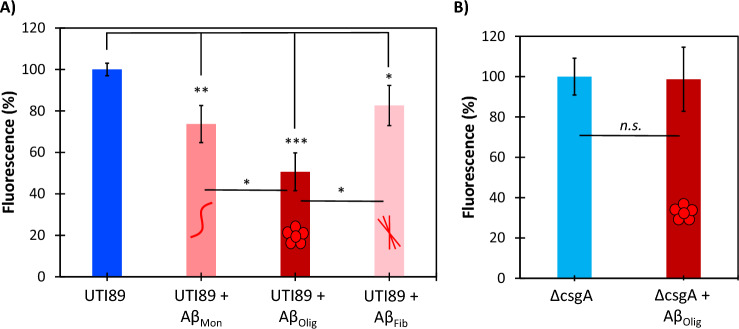
Aβ oligomers inhibit amyloid formation in UTI89. (**A**) Incubation with 7.5 µM Aβ oligomers (0.5 pg/CFU) caused a 50% reduction of curli production by UTI89 (*p* = 0.0009). Monomeric and fibrillar Aβ caused 26% and 17% curli inhibition, respectively (*p* = 0.008 and *p* = 0.04). (**B**) Incubation with 7.5 µM Aβ oligomers (0.5 pg/CFU) had no effect on the ThT signal of ∆csgA. The monomeric, α-sheet oligomer, and β-sheet fibril samples of Aβ are labeled as Aβ_Mon_, Aβ_Olig_, Aβ_Fib_, respectively.

Using a protocol incorporating a Thioflavin T (ThT) assay for measuring Aβ aggregation and β-sheet formation, circular dichroism spectroscopy (CD) for determining secondary structure content, and cell viability assays^3^, we isolated Aβ enriched in its three conformations: nontoxic monomeric random coil; toxic oligomeric α-sheet; and nontoxic fibrillar β-sheet (SI Fig. 1). UTI89 and UTI89 ∆csgA strains were grown in biofilm-forming conditions with the Aβ samples to measure the effect of Aβ structure on curli formation. Curli formation was inhibited by each Aβ sample, with the most effective inhibition observed in UTI89 biofilms grown with the α-sheet oligomeric form of Aβ (50% reduction in ThT signal) (Fig. 4A). Biofilm amyloid content was reduced 26% and 17% by monomeric and fibrillar Aβ, respectively (Fig. 4A). Oligomeric Aβ had no effect on the ThT fluorescence of UTI89 ∆csgA biofilms (Fig. 4B).

### Aβ oligomers inhibit *E. coli* biofilm formation

**Figure 5:**
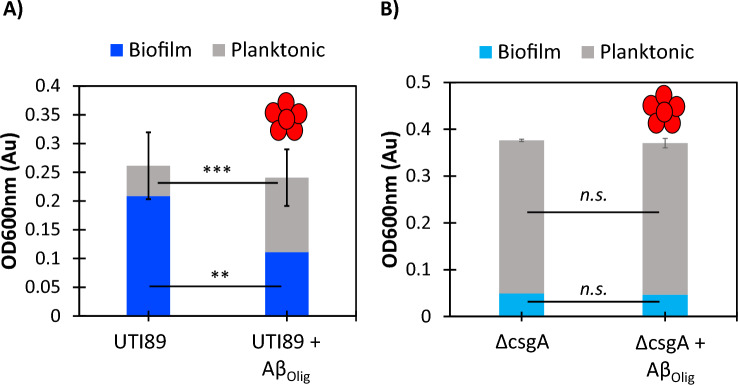
Optical density measurements indicate that Aβ oligomers reduce UTI89 biofilm cell density but do not cause cell death. (**A**) Incubation with 7.5 µM Aβ oligomers (0.5 pg/CFU) reduced UTI89 biofilm cell density by 47% (*p* = 0.008) and increased planktonic cell density by 150% (*p* = 0.0003). Aβ oligomers had no significant effect on the total cell density of UTI89. (**B**) Incubation with 7.5 µM Aβ oligomers (0.5 pg/CFU) had no effect on the cell dispersion or total cell density of ∆csgA, as measured by OD600. The α-sheet oligomer sample of Aβ is labeled as Aβ_Olig_.

In addition to measuring the amyloid content in UTI89 biofilms, we also measured optical density at 600 nm (OD_600_) to determine the relative cell and biofilm density among the various conditions. UTI89 biofilms grown with 7.5 µM oligomeric Aβ (0.5 pg/CFU) exhibited a 47% reduction in cell and biofilm density (Fig. 5A). Notably, the density of planktonic, free-floating cells increased 1.5-fold. The difference in total cell density (planktonic + biofilm) as measured by OD_600_ between the UTI89 and UTI89 + Aβ conditions was not statistically significant (p > 0.05), reflecting the shift from cells in the biofilm to planktonic phase. Aβ had no effect on the cell density distribution of the non-amyloid forming ∆csgA strain (Fig. 5B).

### Aβ oligomers improve *E. coli* antibiotic susceptibility

**Figure 6:**
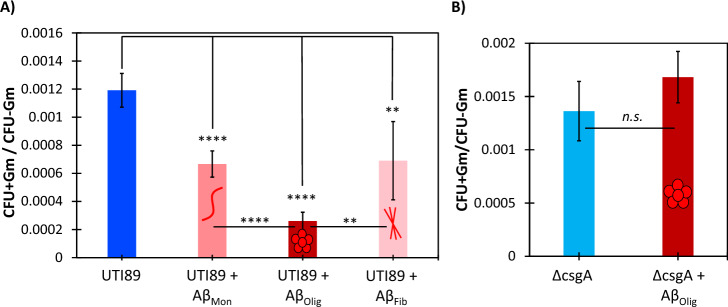
Aβ α-sheet oligomers increase UTI89 susceptibility to gentamicin. Ratios of CFU of *E. coli* + gentamicin/*E. coli − *gentamicin were calculated for bacteria grown in the presence and absence of 7.5 µM Aβ (0.5 pg/CFU) and compared. (**A**) Aβ oligomers increased UTI89 susceptibility to gentamicin 79% (*p* = 3 × 10^–8^). Monomeric and fibrillar Aβ increased UTI89 susceptibility to gentamicin 44% and 42%, respectively (*p* = 0.00002 and *p* = 0.004). (**B**) Aβ oligomers decreased ∆csgA susceptibility to gentamicin 23%, but the difference is not statistically significant. The monomeric, α-sheet oligomer, and β-sheet fibril samples of Aβ are labeled as Aβ_Mon_, Aβ_Olig_, Aβ_Fib_, respectively.

We then conducted antibiotic susceptibility experiments to determine whether amyloid and biofilm inhibition by Aβ oligomers results in increased susceptibility of the bacteria to antibiotics. Because biofilm cells are 10–1000 × less susceptible to antibiotics than planktonic cells^52^, we hypothesized that by inhibiting curli formation and reducing biofilm cell density, Aβ oligomers would increase the susceptibility of *E. coli* biofilms to antibiotics. We found that all three Aβ samples increased the susceptibility of UTI89 biofilms to gentamicin, and oligomeric Aβ had the largest effect corresponding to a 79% increase in susceptibility (Fig. 6A). Monomeric and fibrillar Aβ increased UTI89 biofilm susceptibility to gentamicin by 44% and 42%, respectively (Fig. 6A). and ∆csgA did not have an effect on susceptibility to gentamicin (Fig. 6B).

### CsgA and Aβ toxic oligomers neutralize each other

**Figure 7:**
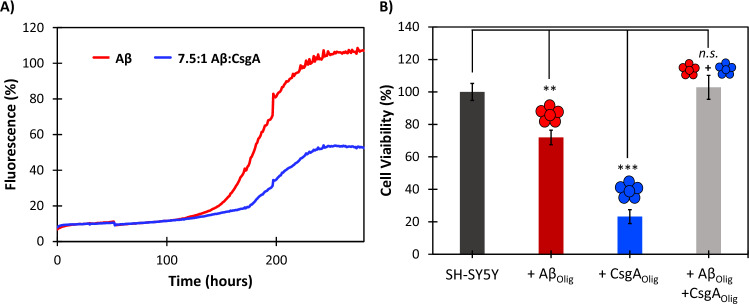
Aβ and CsgA oligomers interact in vitro*. (A*) CsgA inhibits Aβ aggregation 47% (*p* = 0.03). Inhibition begins during the late lag phase of aggregation when oligomers are formed. (**B**) Aβ and CsgA oligomers reduce SH-SY5Y neuroblastoma cell viability 28% and 77%, respectively (*p* = 0.002 and *p* = 0.0004). Co-administration of Aβ and CsgA oligomers results in complete recovery of cell viability (*p* = 0.64). The α-sheet oligomer samples of Aβ are labeled as Aβ_Olig_ and those of CsgA are labeled CsgA_Olig_.

To further investigate the molecular interactions that govern *E. coli* amyloid and biofilm inhibition by Aβ, we conducted in vitro aggregation assays with CsgA and Aβ. CsgA inhibited the aggregation of Aβ (excess Aβ 7.5:1) by approximately 43% (Fig. 7A). Notably, the ThT fluorescence signal of Aβ began to separate from the coincubation fluorescence at the end of the lag phase when oligomeric conformers are dominant just prior to conversion to β-sheet (Fig. 7A). Binding of the CsgA oligomers extended the lag from approximately 150 to 175 h. Further details for the structural changes observed by Aβ and CsgA, separately, during amyloidogenesis are provided in Shea et al.^3^ and Bleem et al.^43^, respectively. Cell toxicity experiments with oligomeric Aβ and CsgA were also conducted to determine whether the amyloid proteins could neutralize one another’s oligomeric toxicity. Oligomeric Aβ and CsgA samples rich in oligomeric α-sheet conformers were isolated, as described previously by Shea et al.^3^ (Aβ) and Bleem et al.^43^ (CsgA) (SI Fig. 1). The Aβ oligomers reduced the cell viability of SH-SY5Y human neuroblastomas by 28%, and CsgA reduced the cell viability by 77% (Fig. 7B). Co-incubation of Aβ and CsgA oligomers (excess Aβ 7.5:1) led to complete recovery of cell viability.

## Discussion

### Aβ α-sheet oligomers promote *E. coli* biofilm clearance

The results reported here provide mechanistic insights into the role of Aβ in the innate immune response. Neuroblastoma cells upregulate the production of Aβ oligomers when grown with UTI89, but not with the non-amyloid forming control strain, ∆csgA (Fig. 3). This suggests that Aβ forms toxic oligomers when exposed to bacterial amyloid, likely to protect against the invading pathogen. As shown in Fig. 4A, Aβ oligomers inhibited UTI89 curli biogenesis but did not affect the ThT fluorescence of the ∆csgA control strain (Fig. 4B). Comparison of the results described in Fig. 4A,B confirms that the decreased ThT signal observed with the UTI89 strain is due to curli inhibition rather than any signal reducing effect by the Aβ oligomers. Further, Aβ oligomers weakened UTI89 biofilm formation but did not promote cell death (Fig. 5A). Instead, curli and biofilm inhibition by Aβ oligomers shifted cells from the biofilm to the free-floating planktonic phase. As planktonic cells are more susceptible to clearance by the outside environment (i.e., antibiotics and host immune response), bacterial amyloid inhibition by Aβ likely serves to weaken the pathogen to promote clearance by immune cells. This non-bactericidal activity corresponds to previous studies indicating that de novo α-sheet peptides inhibit amyloid formation and reduce biofilm cell density without promoting cell death^43^. Additionally, Aβ oligomers had no effect on the relative cell distribution between the planktonic and biofilm phases of the UTI89 ∆csgA control strain (Fig. 5B), suggesting that UTI89 curli and biofilm inhibition by Aβ oligomers is due to specific interactions between CsgA and Aβ.

The observed increase in gentamicin susceptibility by UTI89 (Fig. 6A) when grown in the presence of toxic Aβ oligomers also supports the data reported in Figs. 4A and 5A, as well as the results obtained from previous experiments conducted with uropathogenic *E. coli* and de novo α-sheet peptides^43^. In our previous study, we also showed that curli inhibition by de novo α-sheet peptides increased susceptibility to gentamicin and promoted macrophage clearance of uropathogenic *E. coli*^43^. We hypothesize that curli and biofilm inhibition by Aβ may promote similar macrophage clearance. Notably, curli inhibition and elevated *E. coli* susceptibility to gentamicin was observed by all Aβ samples, with the largest effect attributed to the sample enriched in the toxic, oligomeric α-sheet conformation. As previously mentioned, mixed populations are present throughout the early stages of amyloidogenesis prior to the deposition of stable β-sheet fibrils (Fig. 1). As Aβ converts from random coil to α-sheet oligomers, both species are present, and the toxic oligomers become the dominant species during the late lag period (Fig. 1, and as demonstrated and discussed by Shea et al.^3^). These oligomers are a mix of hexamers and dodecamers^3^. Similarly, during the exponential phase and early plateau phase (Fig. 1), both α-sheet oligomers and β-sheet protofibrils co-exist^3^. Consequently, the effects observed by the “monomeric” and “fibrillar” Aβ species are likely due to a minor presence of α-sheet oligomers.

Finally, the results reported in Fig. 7 provide insight into the molecular mechanisms that may govern the inhibitory properties of Aβ oligomers. The ThT data indicate that amyloid inhibition occurred during the late lag phase of aggregation, when Aβ and CsgA oligomers are present^3,43^. Additionally, the toxicity of both the Aβ and CsgA oligomers was neutralized when they were co-administered to human SH-SY5Y neuroblastoma cells. The data reported in Fig. 7 suggest that Aβ oligomers inhibit curli and biofilm formation by UTI89 via specific interactions between the toxic α-sheet oligomers of both species. Interestingly, the CsgA oligomers were significantly more toxic to the human SH-SY5Y neuroblastoma cells than the Aβ oligomers. This may be due to a higher proportion of α-sheet structure in the CsgA sample relative to the Aβ sample due to the inherent heterogeneity of oligomeric samples. Alternatively, an inherent cross-species response to the CsgA oligomers by the human neuroblastomas may cause an increased toxicity response.

While we focus here on the interactions between Aβ and α-sheet oligomers on amyloid formed by *E. coli*, we hypothesize that Aβ oligomers promotes immune clearance of many microbial species, including other amyloid-forming bacteria as well as viruses. A study by Serwer et al. demonstrated that the viral capsids produced by herpes virus are rich in α-sheet structure, and they hypothesize that the presence of α-sheet structure in the capsids promotes the production of Aβ α-sheet oligomers and subsequent neurodegeneration^53^. Therefore, it is possible that the colocalization of Aβ plaques and HSV1, as reported in multiple studies^11,16,54^, may be a result of specific interactions between Aβ α-sheet oligomers and the viral capsids produced by herpes virus that contain α-sheet structure.

### AD: an infectious disease?

It was previously believed that microbes entered the brain only during severe brain infections; however, a growing body of evidence suggests that various bacteria, viruses, and fungi reside even in healthy brains. The electronic tree of life (eToL) developed by Lathe et al. uses small subunit ribosomal RNA (rRNA) probes to investigate the diversity of microorganisms in both control and AD brain samples^41,55^. An abundance of bacteria, fungi, and chloroplastida were identified in both control and AD brains, and the brain microbiome was reported to contain approximately ~ 20% of the diversity of the gut microbiome^41^. Notably, the spectrum of microorganisms found in the brain varied significantly between individuals, and this observed diversity is likely due to variations between environmental exposure and genetic predisposition^41^. Microorganism diversity also varied between brain regions, and there was evidence of pathogens spreading between brain regions in single individuals^41^. Certain microbial species were over-represented in AD brain samples including *Streptococus*, *Staphylococcus*, *Altenaria*, and *Cortinarius*, suggesting that while many microorganisms may reside safely in the brain, others are more likely to trigger Aβ aggregation and AD pathology^41^. Interestingly, the concentration of brain microbes significantly increased with age^41^, which may be due, in part, to Aβ clearance rate decreasing with age^56^, as well as other AD risk factors such as increased blood pressure and cognitive decline that are often associated with aging. And, in the case of AD, microorganisms in the brain are further elevated due to breakdown of the blood brain barrier due to chronic inflammation from the accumulation of toxic Aβ aggregates^57^.

The classification of AD as an infectious disease provides insight into potential prevention and therapeutic strategies. There is typically a 10–30-year window in which Aβ aggregates accumulate and trigger the formation of tau tangles prior to the onset of cognitive symptoms^10,57,58^. Proper treatment of microbial infection and reducing chronic inflammation during this time may aid in reducing neuronal death and delaying disease onset, particularly in individuals genetically predisposed to AD. Our research suggests that the presence of amyloid-forming microorganisms may trigger upregulation of “protective” toxic Aβ oligomers. The data reported here also suggest that Aβ oligomers specifically inhibit curli formation via complementary α-sheet interactions between CsgA and Aβ oligomers, weaken *E. coli* biofilms, and inhibit CsgA oligomer toxicity, thereby identifying a molecular mechanism through which Aβ acts as an antimicrobial peptide.

## Conclusions

Numerous studies have provided evidence for a probable connection between microbial infection and AD, including the role of Aβ in the innate immune response. However, little is known about the molecular mechanisms involved in the aggregation of Aβ as an immune response. This study investigates the capacity for Aβ to inhibit *E. coli* biofilm formation by preventing the formation of curli and suggests that the same oligomeric species that causes neuronal cell death in AD serves a protective function against infection. We hypothesize that Aβ oligomers may inhibit amyloid formation of several amyloid-forming pathogens, including bacteria, viruses, and fungi, through interactions involving α-sheet oligomers.

## Materials and methods

### Neuroblastoma and uropathogenic *E. coli* co-incubation

SH-SY5Y human neuroblastomas (American Type Culture Collection; Manassas, VA) were cultured in 1:1 DMEM:F12 (Invitrogen; Carlsbad, CA) supplemented with 10% FBS (Invitrogen; Carlsbad, CA), 100 units/mL penicillin (Invitrogen; Carlsbad, CA), 100 µg/mL streptomycin (Invitrogen; Carlsbad, CA). The cells were seeded in a 48-well sterile tissue culture-treated plate (Corning; Glendale, AZ) at 2.4 × 10^5^ cells per well and cultured in CO_2_ water-jacketed incubator (37 °C, 5% CO_2_; Thermo Fisher Scientific; Waltham, MA) for 24 h. A uropathogenic clinical isolate strain, UTI89^59^, and a control strain with a chromosomal deletion of the CsgA gene, UTI89 ∆csgA^60^, were used for all *E. coli* experiments. Overnight cultures were grown in Luria Broth (LB, Miller, Thermo Fisher Scientific; Waltham, MA) for 16–18 h at 37 °C with shaking (180 rpm). Cultures were then “refreshed” by replacing 5 mL of culture with 5 mL fresh LB medium and grown for an additional three hours to ensure bacteria were in the exponential phase. Overnight cultures were then diluted to an optical density (OD_600_) of 0.3 (~ 2.4 × 10^8^ cells/mL) in YESCA broth supplemented with 4% DMSO (Corning; Glendale, AZ), medium known to promote increased curli formation^61^. The two bacterial suspensions were diluted threefold in neuroblastoma cell media without antibiotics (1:1 DMEM:F12 supplemented with FBS). A vehicle control condition was prepared with YESCA, DMSO, and DMEM:F12 at the identical ratio. After neuroblastoma cells had seeded for 24 h, media was removed and replaced with 100 µL of vehicle control, UTI89, or UTI89 ∆csgA. Each of the three conditions were plated in triplicate. The samples were grown for 48 h at 26 °C to promote biofilm formation. Cells were monitored consistently to ensure that the reduced temperature did not result in neuroblastoma cell death. The supernatant was then removed from each well and applied to SOBA for Aβ oligomer quantification.

### Soluble oligomer binding assay

The SOBA assay was conducted according to a modified protocol previously discussed in Shea et al.^50^ with a plate washer incorporated for several of the wash steps. Dopamine HCl (Sigma-Aldrich; St. Louis, MO) was dissolved into Tris Buffer Saline-Tween (TBS-T) (pH 7.4, 50 mM tris, 100 mM NaCl, 0.01% Tween-20) at 10 mg/mL. 150 μL of the dopamine solution was added to each well of an opaque Nunc Immobilizer Amino 96-well plate (Corning; Corning, NY) and shaken at 340 rpm at room temperature for 20–24 h covered in foil. A plate washer was then primed with 1 L H_2_O, and the 96-well plate was aspirated and washed with 100 µL H_2_O 10 times. The plate washer was flushed, and the manifold sonicated for 90 min, and the 96-well plate was dried in a 37 °C incubator for 1 h. α-Sheet peptide (AP193^3,43,50^) was dissolved in dimethyl sulfoxide (Sigma Aldrich; St. Louis, MO) to 36 mM, and diluted to 60 μM in carbonate buffer (pH 9.6, 100 mM CO_3_^2−^). The α-sheet peptide solution was incubated in a 37 °C water bath for 1 h to dimerize. 100 μL of 60 μM AP193 in carbonate buffer was then added to each well of the 96-well plate, and the plate was shaken at 340 rpm at room temperature for 1 h, or up to 2 h to couple the peptide to the 96-well plate. The plate was then aspirated and washed with PBS-T (pH 7.4, 137 mM NaCl, 2.7 mM KCl, 10 mM Na_2_HPO_4_, 1.8 mM KH_2_PO4, 0.05% Tween-20) 3 times. The washer was then flushed with 1 L H_2_O and the manifold sonicated for 30 min. 150 μL of 10 mM ethanolamine (Sigma Aldrich; St. Louis, MO) in carbonate buffer was added to each well, and the 96-well plate was shaken at 340 rpm at room temperature for 2 h while covered in foil to quench unreacted sites in each well. The plate was then washed with PBS-T 3 times, and the plate washer flushed with 1 L H_2_O and the manifold sonicated for 30 min. 300 μL Pierce Protein-Free Blocking Buffer (Thermo Fisher Scientific; Waltham, MA) was added to each well of the plate and decanted by inverting the plate 3 times. Then, 100 μL of each neuroblastoma *E. coli* co-incubation sample was applied to the surface of each well and incubated at 1 h at 25 °C without shaking. The plate washer was then primed with 2 L H_2_O. The 96-well plate was then aspirated and washed with PBS 3 times, the plate washer was flushed with 1 L H_2_O, and the manifold sonicated for 30 min. 100 μL of 0.03 μg/mL of 6E10 HRP anti-β-Amyloid (BioLegend; San Diego, CA) dissolved in 3% BSA in TBS-T was added to each well, and the plate was shaken at 340 rpm for 1 h, covered in foil. The plate washer was primed with 1 L H2O. Then, the plate was aspirated and washed with PBS 3 times, and the plate washer was flushed with 1 L H_2_O and the manifold sonicated for 1.5 h. 115 μL of room temperature SuperSignal ELISA Femto 9 Maximum Sensitivity Substrate (Thermo Fisher Scientific; Waltham, MA) was plated per well, and the plate was shaken for 1 min covered in foil before reading the luminescence in a Tecan plate reader (Mannendorf, Switzerland) with a 0.2 s integration time. Chemiluminescence readings were obtained within 15 min for maximum signal.

### Aβ stock preparation

Aβ (1–42), referred to as Aβ, was obtained from ERI Amyloid Laboratory, LLC (Oxford, CT) and stock solution was prepared as discussed previously^3^. Aβ was solubilized using hexafluoroisopropanol (HFIP, Sigma-Aldrich; St. Louis, MO) to 1 mg/mL. The Aβ solution was sonicated in a bath sonicator for 5 min, then incubated on ice for 25 min. The sonication and icing incubation process was repeated a second time. The HFIP was then evaporated under a gentle N_2_ stream, and the Aβ was concentrated using a SpeedVac concentrator (Savant ISS110, Thermo Fisher Scientific; Waltham, MA) for two hours on low setting. The monomerized Aβ film was stored at − 20 °C until use. Aβ stock was prepared by removing the aliquoted film from − 20 °C and allowing it to equilibrate to room temperature (RT) for approximately 5 min. The film was dissolved to 0.75 mg/mL in 6 mM NaOH (pH 11.6, Sigma-Aldrich; St. Louis, MO) solution and sonicated in 5-min intervals until fully solubilized. The solution was centrifuged at 7000 rpm for 2 min in a 0.22 µm Costar Spin-X cellulose acetate centrifuge filter (Sigma-Aldrich; St. Louis, MO) to remove any remaining seeds. The solution was then transferred to an Eppendorf LoBind microcentrifuge tube (Sigma-Aldrich; St. Louis, MO) and concentration was measured using a NanoDrop 2000 Spectrometer (Thermo Fisher Scientific; Waltham, MA) at 280 nm using an extinction coefficient of 1490 M^−1^ cm^−1^. The stock solution was incubated at 25 °C for 4 h to promote complete monomerization, as confirmed by size exclusion chromatography^3^, and either used immediately or stored for up to 1 week at 4 °C.

### Aβ aggregation for biofilm inhibition experiments

Aβ samples enriched in each conformation (nontoxic random coil monomer, soluble toxic α-sheet oligomer, and nontoxic β-sheet fibril) were isolated for *E. coli* biofilm and curli inhibition experiments. “Undisturbed” aggregation experiments were conducted as described previously^3^. Stock Aβ was aliquoted into Eppendorf LoBind microcentrifuge tubes (Sigma-Aldrich; St. Louis, MO) and diluted using phosphate buffered saline (PBS) buffer (10 mM phosphate, 130 mM NaCl, and 2.7 mM KCl; Sigma-Aldrich, St. Louis, MO) to 75 µM. One tube was prepared for each time point that was to be measured by ThT. First, a portion of the stock Aβ was aliquoted into a LoBind tube. PBS buffer was then added gently to the side of this tube for a final volume of 185 µL and final concentration of 75 µM and mixed 3× by pipette. Aliquots were incubated at 25 °C. At the desired time point, a single tube was removed from the incubator for measurement by ThT. Concentrated stock ThT (Thermo Fisher Scientific; Waltham, MA) was prepared monthly, by dissolving ThT powder to 5 mg/mL in H2O; concentration was measured of 1:10 dilutions of the stock using a NanoDrop 2000 Spectrometer at 412 nm using an extinction coefficient of 36,000 M^−1^ cm^−1^. Concentrated ThT stock was added to the Aβ solution, with the calculated volume corresponding to a final concentration of 24 µM ThT for aggregation studies (typically ~ 1.0 µL). The resulting solution was gently mixed once by pipette, and 60 µL was added to a single well (in triplicate) in a black 384-well plate and read on a multimode plate reader (PerkinElmer; Waltham, MA). Relevant parameters include: λ_ex_ 438 nm, λ_em_ 495 nm, measurement height 7.5 mm, 8 flashes.

### Circular dichroism spectroscopy

Circular dichroism (CD) studies were conducted as described previously^3^. Pre-incubated Aβ at 75 µM (timepoints determined by SI Fig. 1) was diluted in PBS to a concentration of 25 µM for CD. A portion of the peptide solution was added, first, to a LoBind tube. The corresponding volume of buffer was then added gently—along the side of the tube—to the peptide-containing LoBind tube and mixed once by pipette to ensure adequate mixing. 300 µL of the resulting solution was added to a 1 mm pathlength quartz cuvette (Starna Cells; Atascadero, CA) and scans collected using a Jasco J-720 CD machine. All experiments aggregated 8 scans and used a Savitzky-Golay smoothing protocol, followed by reduction of noise by an FFT filter. The mean residual ellipticity (MRE) was calculated by first subtracting the peptide signal from the blank signal, and the curve zeroed against the value at 270 nm.

### *E. coli* biofilm growth

Overnight cultures of UTI89 and UTI89 ∆csgA were prepared as described above, then diluted to an optical density (OD_600_) of 0.1 (~ 8 × 10^7^ cells/mL) in YESCA broth supplemented with 4% DMSO (Corning; Glendale, AZ), medium known to promote increased curli formation^61^. Diluted bacteria culture (180 µL) was plated with 20 µL Aβ (or NaOH/PBS, in the case of controls) and aliquoted in triplicate into wells of a sterile, clear 48-well polystyrene plate (Corning; Glendale, AZ). Aβ was pre-incubated as described above for 0, 30, and 72 h (time points chosen based on SI Fig. 1 to correspond to random coil, α-sheet, and β-sheet, respectively^3^. The final Aβ concentration was 0 or 7.5 µM (0.5 pg/CFU). Plates were covered, sealed in a plastic bag, and grown at 26 °C for 48 h without shaking.

Planktonic cells and medium were then removed, and biofilms were rinsed once with 250 μL PBS. Planktonic cells were spun down and resuspended in PBS, and the optical density of the planktonic samples was determined at 600 nm to estimate planktonic cell density. The PBS rinse solution was removed from the wells and biofilms were resuspended in 200 μL of 20 μM ThT in PBS (Sigma-Aldrich, St. Louis, MO). Biofilms were homogenized by vigorous pipetting (30× per well), 3 min of sonication, and 1 min on a plate shaker. 100 μL of each biofilm suspension was then transferred to a black-walled, clear-bottom 96 well plate for measurements in a plate reader (PerkinElmer, Waltham, MA). ThT fluorescence was measured at 438/495 nm as a proxy for amyloid formation, and biofilm absorbance was measured at 600 nm to estimate bacterial cell density. Each pg/CFU calculation was done by assuming that OD_600_ = 0.1 corresponds to 8 × 10^7^ cells/mL.

### Antibiotic susceptibility

Biofilms were grown according to the methods described above. Gentamicin sulfate (Thermo Fisher Scientific; Waltham, MA) was dissolved in YESCA at a concentration of 900 µg/mL. 100 µL of antibiotic or control (YESCA media) was added to each well after 42 h of incubation without disturbing the biofilm for a final gentamicin concentration of 300 µg/mL. Following 6 additional hours of biofilm growth (48 total), planktonic cells were removed and discarded. The biofilms were rinsed with 250 µL PBS and the rinse was discarded. Biofilms were homogenized in 200 µL PBS by vigorous pipetting (30× per well), and the biofilm suspensions were transferred to an Eppendorf tube. The biofilm suspensions were then ultra-sonicated for 5 s on ice and diluted in tenfold increments. The serial dilutions were then plated on agar plates (LB agar) using the drop plate method^62^. Six replicates were plated per condition. Colonies were grown for 16 h at 37 °C and CFUs were counted. Total CFUs of the biofilm suspensions were calculated using the dilution number and the number of CFUs counted in that dilution. CFU+Gm/CFU-Gm ratios were calculated by dividing the values of each of the six CFU+ gentamicin replicates by the average CFU− gentamicin value and then taking the average of the ratios.

### CsgA purification

A synthetic gene corresponding to the *E. coli* CsgA protein was designed and synthesized by Addgene (Watertown, MA). The gene was cloned into the pET-22b(+) vector, which added a C-terminal 6× His tag for purification. Plasmids were transformed into *E. coli* BL21 (DE3) cells and protein expression was carried out in 2 L shake flasks at 37 °C. Cultures grew to an OD600nm of 0.6–0.8 prior to induction with 1 mM IPTG. After 3–4 h of additional growth, cells were harvested by centrifugation and resuspended in 30 mL denaturing buffer (8 M Gnd-HCl (Thermo Fisher Scientific; Waltham, MA), 50 mM NaPi (Sigma-Aldrich; St. Louis, MO), pH 8.0) and lysed overnight with stirring at 4 °C. Insoluble material was removed by centrifugation at 14,000 xg for 30 min and 15 mL of supernatant was incubated with 5 mL HisPur Ni–NTA beads (Thermo Fisher; Waltham, MA) for 2 h at room temperature with end-over-end rotation. The beads were then washed twice with denaturing buffer, twice again with denaturing buffer plus 15 mM imidazole (Sigma-Aldrich; St. Louis, MO), and twice again with denaturing buffer plus 30 mM imidazole. Finally, protein was eluted with denaturing buffer plus 400 mM imidazole. Samples from each step of the purification were precipitated from guanidinium hydrochloride by trichloroacetic acid (Thermo Fisher Scientific; Waltham, MA)^63^ and analyzed by SDS-PAGE.

### CsgA and Aβ aggregation studies

CsgA aliquots were desalted immediately prior to use using the Zeba desalting column (7 k MWCO) protocol (Thermo Fisher; Waltham, MA) into buffer (10 µM KCl + 10 µM NaPi, pH 7.4), transferred to an Eppendorf LoBind microcentrifuge tube (Sigma-Aldrich; St. Louis, MO), and kept on ice. Protein concentration was determined by NanoDrop 2000 Spectrometer (Thermo Fisher Scientific; Waltham, MA) at 280 nm using an extinction coefficient of 11,460 M^−1^ cm^−1^. CsgA was aliquoted into a separate LoBind tube (volume calculated to achieve 10 µM concentration in 185 µL). Stock Aβ was prepared as described above and slowly added to the side of the CsgA-containing tube (volume calculated to achieve 75 µM concentration in 185 µL). KCl + NaPi buffer supplemented with 24 µM ThT was gently added to the side of the tube (volume calculated to achieve final volume of 185 µL). The resulting solution was gently mixed 3× by pipette, and 60 µL was added to a single well (in triplicate) in a black 384-well plate and read on a multimode plate reader (Tecan; Mannendorf, Switzerland). Relevant parameters include: λ_ex_ 438 nm, λ_em_ 495 nm, measurement height 7.5 mm, 8 flashes.

### CsgA and Aβ cellular toxicity studies

Cell viability was determined using a 3-(4,5-dimethylthiazol-2-yl)-2,5-diphenyltetrazolium bromide (MTT) assay^64^. SH-SY5Y human neuroblastomas were seeded at 2.4 × 10^5^ cells per well in a 48-well tissue culture-treated plate and incubated for 24 h at 37 °C and 5% CO_2_ as described above. CsgA (10 µM) and Aβ (75 µM) were prepared as described above and incubated both separately and together in KCl + NaPi buffer at 25° until they reached the late lag phase of aggregation (t = 150 h), as informed by Fig. 7A. After 24 h of cell seeding, the cell culture media was removed and replaced with 100 µL preincubated Aβ, CsgA, or Aβ + CsgA (or NaPi + KCl, in the case of controls) diluted 1:3 in cell media. The cells were cultured with experimental solution for 24 h at 37 °C before addition of 25 µL MTT (5 mg/mL in PBS; Sigma-Aldrich), then incubated for 4 h at 37 °C. 100 µL lysis buffer (50% DMF, 20% SDS, 1% glacial acetic acid, and 0.2% HCl) was added to each well and incubated overnight at 25 °C covered in foil. The optical density was read at 570 nm with a multimode plate reader (Tecan; Mannendorf, Switzerland).

### Statistics

All statistical significance values reported were calculated using a two-tailed T-test. A single asterisk indicates a p value less than 0.05. Two asterisks indicate a p value less than 0.01. Three asterisks indicate a p value less than 0.001. Four asterisks indicate a p value less than 0.0001.

### Supplementary Information

Supplementary Information.

## Data Availability

The datasets used and/or analyzed during the current study are available from the corresponding author on reasonable request.

## References

[1] Alzheimer’s Association (2016). 2016 Alzheimer’s disease facts and figures. Alzheimer’s Dementia.

[2] Kelley BJ, Petersen RC (2007). Alzheimer’s disease and mild cognitive impairment. Neurol. Clin..

[3] Shea D (2019). α-Sheet secondary structure in amyloid β-peptide drives aggregation and toxicity in Alzheimer’s disease. Proc. Natl. Acad. Sci. USA.

[4] Yang T, Li S, Xu H, Walsh DM, Selkoe DJ (2017). Large soluble oligomers of amyloid β-protein from alzheimer brain are far less neuroactive than the smaller oligomers to which they dissociate. J. Neurosci..

[5] Wang J, Dickson DW, Trojanowski JQ, Lee VM-Y (1999). The levels of soluble versus insoluble brain Aβ distinguish Alzheimer’s disease from normal and pathologic aging. Exp. Neurol..

[6] Haass C, Selkoe DJ (2007). Soluble protein oligomers in neurodegeneration: Lessons from the Alzheimer’s amyloid beta-peptide. Nat. Rev. Mol. Cell Biol..

[7] McLean CA (1999). Soluble pool of Aβ amyloid as a determinant of severity of neurodegeneration in Alzheimer’s disease. Ann. Neurol..

[8] Lathe R (2023). Establishment of a consensus protocol to explore the brain pathobiome in patients with mild cognitive impairment and Alzheimer’s disease. Alzheimer’s Dementia.

[9] Itzhaki RF (2016). Microbes and Alzheimer’s disease. J. Alzheimer’s Dis..

[10] Jorfi M, Maaser-Hecker A, Tanzi RE (2023). The neuroimmune axis of Alzheimer’s disease. Genome Med..

[11] Eimer WA (2018). Alzheimer’s disease-associated β-amyloid is rapidly seeded by herpesviridae to protect against brain infection. Neuron.

[12] Soscia SJ (2010). The Alzheimer’s disease-associated amyloid β-protein is an antimicrobial peptide. PLoS One.

[13] Kumar DKV (2016). Amyloid-β peptide protects against microbial infection in mouse and worm models of Alzheimer’s disease. Sci. Transl. Med..

[14] Allen HB (2016). Alzheimer’s disease: Assessing the role of spirochetes, biofilms, the immune system, and amyloid-β with regard to potential treatment and prevention. J. Alzheimer’s Dis..

[15] Miklossy J (2011). Alzheimer’s disease—a neurospirochetosis. Analysis of the evidence following Koch’s and Hill’s criteria. J. Neuroinflamm..

[16] Wozniak M, Mee A, Itzhaki R (2009). Herpes simplex virus type 1 DNA is located within Alzheimer’s disease amyloid plaques. J. Pathol..

[17] Zhan X (2016). Gram-negative bacterial molecules associate with Alzheimer disease pathology. Neurology.

[18] Dominy SS (2019). Porphyromonas gingivalis in Alzheimer’s disease brains: Evidence for disease causation and treatment with small-molecule inhibitors. Sci. Adv..

[19] Cribbs DH (2012). Extensive innate immune gene activation accompanies brain aging, increasing vulnerability to cognitive decline and neurodegeneration: A microarray study. J. Neuroinflamm..

[20] McGreer P, McGreer E (1995). The inflammatory response system of brain: Implications for therapy of Alzheimer and other neurodegenerative diseases. Brain Res. Rev..

[21] Akiyama H (2000). Inflammation and Alzheimer’s disease. Neurobiol. Aging.

[22] Akama KT, Van Eldik LJ (2000). β-amyloid stimulation of inducible nitric-oxide synthase in astrocytes is interleukin-1β- and tumor necrosis factor-α (TNFα)-dependent, and involves a TNFα receptor-associated factor- and NFκB-inducing kinase-dependent signaling mechanism. J. Biol. Chem..

[23] Walters A, Phillips E, Zheng R, Biju M, Kuruvilla T (2016). Evidence for neuroinflammation in Alzheimer’s disease. Prog. Neurol. Psychiatry.

[24] Tuppo EE, Arias HR (2005). The role of inflammation in Alzheimer’s disease. Int. J. Biochem. Cell Biol..

[25] Mrak RE, Sheng JG, Griffin WST (1995). Glial cytokines in Alzheimer’s disease: Review and pathogenic implications. Hum. Pathol..

[26] Chen W-W, Zhang X, Huang W-J (2016). Role of neuroinflammation in neurodegenerative diseases (Review). Mol. Med. Rep..

[27] McCombe AP, Henderson DR (2011). The role of immune and inflammatory mechanisms in ALS. Curr. Mol. Med..

[28] Wang Q, Liu Y, Zhou J (2015). Neuroinflammation in Parkinson’s disease and its potential as therapeutic target. Transl. Neurodegener..

[29] Herrero M-T, Estrada C, Maatouk L, Vyas S (2015). Inflammation in Parkinson’s disease: Role of glucocorticoids. Front. Neuroanat..

[30] Kinney JW (2018). Inflammation as a central mechanism in Alzheimer’s disease. Alzheimer’s Dementia Transl. Res. Clin. Interv..

[31] Rubio-Perez JM, Morillas-Ruiz JM (2012). A review: Inflammatory process in Alzheimer’s disease, role of cytokines. Sci. World J..

[32] Meraz-Ríos MA, Toral-Rios D, Franco-Bocanegra D, Villeda-Hernández J, Campos-Peña V (2013). Inflammatory process in Alzheimer’s disease. Front. Integr. Neurosci..

[33] Grammas P (2011). Neurovascular dysfunction, inflammation and endothelial activation: Implications for the pathogenesis of Alzheimer’s disease. J. Neuroinflamm..

[34] Ferreira ST, Clarke JR, Bomfim TR, De Felice FG (2014). Inflammation, defective insulin signaling, and neuronal dysfunction in Alzheimer’s disease. Alzheimer’s Dementia.

[35] Hickman SE, Allison EK, El Khoury J (2008). Microglial dysfunction and defective β-amyloid clearance pathways in aging Alzheimer’s disease mice. J. Neurosci..

[36] Meda L (1995). Activation of microglial cells by β-amyloid protein and interferon-γ. Nature.

[37] Mantri S, Shah BB (2016). Enterovirus causes rapidly progressive dementia in a 28-year-old immunosuppressed woman. J. Neurovirol..

[38] Kristoferitsch W (2018). Secondary dementia due to Lyme neuroborreliosis. Wien Klin Wochenschr..

[39] Vargas A, Carod-Artal F, Del Negro M, Rodrigues M (2000). Dementia caused by neurosyphilis: Clinical and neuropsychological follow-up of a patient. Arq. Neuropsiquiatr..

[40] Wiwanitkit V (2014). Dementia and neurocysticercosis. Acta Neurol. Taiwan.

[41] Hu X, Mckenzie C-A, Smith C, Haas JG (2023). The remarkable complexity of the brain microbiome in health and disease. BioRxiv.

[42] Bleem A, Francisco R, Bryers JD, Daggett V (2017). Designed α-sheet peptides suppress amyloid formation in Staphylococcus aureus biofilms. NPJ Biofilms Microbiomes.

[43] Bleem A (2023). Designed α-sheet peptides disrupt uropathogenic *E. coli* biofilms rendering bacteria susceptible to antibiotics and immune cells. Sci. Rep..

[44] Paranjapye N, Daggett VD (2018). Novo designed α-sheet peptides inhibit functional amyloid formation of *Streptococcus mutans* biofilms. J. Mol. Biol..

[45] Kellock J, Hopping G, Caughey B, Daggett V (2016). Peptides composed of alternating L- and D-amino acids inhibit amyloidogenesis in three distinct amyloid systems independent of sequence. J. Mol. Biol..

[46] Hopping G (2014). Designed α-sheet peptides inhibit amyloid formation by targeting toxic oligomers. Elife.

[47] Jain N (2017). Inhibition of curli assembly and *Escherichia coli* biofilm formation by the human systemic amyloid precursor transthyretin. Proc. Natl. Acad. Sci..

[48] Jain, A. & Zahra, F. *Transthyretin Amyloid Cardiomyopathy (ATTR-CM)*. StatPearls Publishing (2023). 34662045

[49] Childers MC, Daggett V (2019). Drivers of α-sheet formation in transthyretin under amyloidogenic conditions. Biochemistry.

[50] Shea D (2022). SOBA: Development and testing of a soluble oligomer binding assay for detection of amyloidogenic toxic oligomers. Proc. Natl. Acad. Sci..

[51] Biancalana M, Koide S (2010). Molecular mechanism of Thioflavin-T binding to amyloid fibrils. Biochim. Biophys. Acta Prot. Proteom..

[52] Guo Y, Song G, Sun M, Wang J, Wang Y (2020). Prevalence and therapies of antibiotic-resistance in *Staphylococcus aureus*. Front. Cell. Infect. Microbiol..

[53] Serwer P, Hunter B, Wright ET (2020). Electron microscopy of in-plaque phage T3 assembly: Proposed analogs of neurodegenerative disease triggers. Pharmaceuticals.

[54] Itzhaki RF (1997). Herpes simplex virus type 1 in brain and risk of Alzheimer’s disease. Lancet.

[55] Hu X, Haas JG, Lathe R (2022). The electronic tree of life (eToL): A net of long probes to characterize the microbiome from RNA-seq data. BMC Microbiol..

[56] Patterson BW (2015). Age and amyloid effects on human central nervous system amyloid-beta kinetics. Ann. Neurol..

[57] Whitson HE (2022). Infection and inflammation: New perspectives on Alzheimer’s disease. Brain Behav. Immun. Health.

[58] Long JM, Holtzman DM (2019). Alzheimer disease: An update on pathobiology and treatment strategies. Cell.

[59] Mulvey MA, Schilling JD, Hultgren SJ (2001). Establishment of a persistent *Escherichia coli* reservoir during the acute phase of a bladder infection. Infect. Immun..

[60] Cegelski L (2009). Small-molecule inhibitors target *Escherichia coli* amyloid biogenesis and biofilm formation. Nat. Chem. Biol..

[61] Lim JY, May JM, Cegelski L (2012). Dimethyl sulfoxide and ethanol elicit increased amyloid biogenesis and amyloid-integrated biofilm formation in *Escherichia coli*. Appl. Environ. Microbiol..

[62] Herigstad, B., Hamilton, M. & Heersink, J. *How to optimize the drop plate method for enumerating bacteria*. *Journal of Microbiological Methods* vol. 44 www.elsevier.comrlocaterjmicmeth (2001). 10.1016/s0167-7012(00)00241-4 11165341

[63] Arnold U, Ulbrich-Hofmann R (1999). Quantitative protein precipitation from guanidine hydrochloride-containing solutions by sodium deoxycholate/trichloroacetic acid. Anal. Biochem..

[64] Kumar P, Nagarajan A, Uchil PD (2018). Analysis of Cell viability by the MTT assay. Cold Spring Harb. Protoc..

