# Supplementary material for: Mechanistic insights into the role of amyloid-β in innate immunity
Source: Sci Rep. 2024 Mar 5;14:5376. doi: 10.1038/s41598-024-55423-9 (PMC10912764; doi:10.1038/s41598-024-55423-9)
Supplement: Supplementary file 1 — Supplementary Information. [file 41598_2024_55423_MOESM1_ESM.pdf]

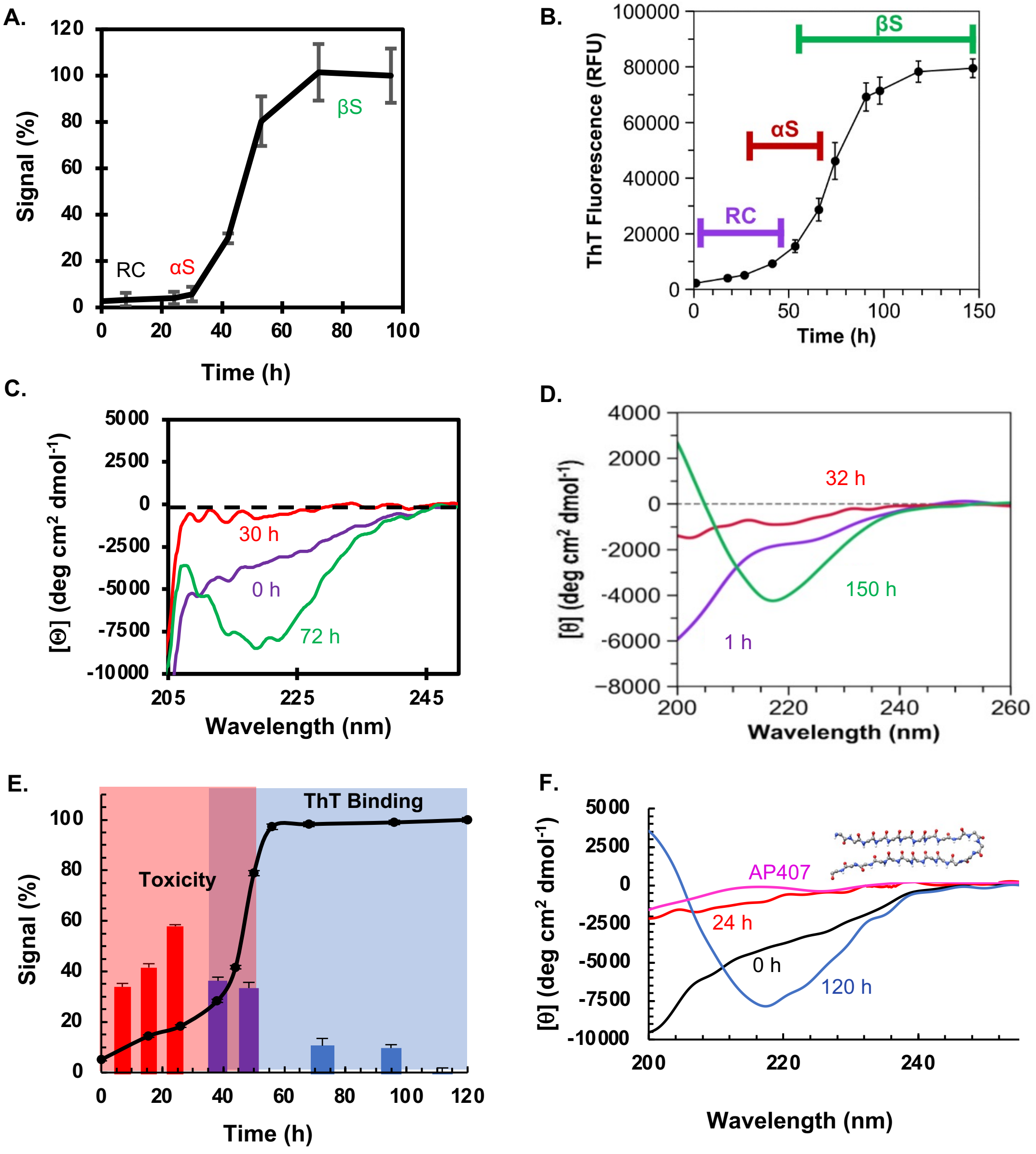

SI Figure 1. Structural characterization of amyloid proteins. A) Undisturbed ThT kinetics of 75 μM Aβ42 indicate that the aggregation lag phase is approximately 30 hours. The lag phase is followed by a rapid increase in ThT signal which plateaus at approximately 72 hours. B) Undisturbed ThT kinetics of 14 μM CsgA indicate that the aggregation lag phase is approximately 50 hours. The lag phase is followed by a rapid increase in ThT signal which plateaus at approximately 120 hours. C) Circular dichroism spectroscopy of three pre-incubated Aβ42 samples (t=0, t=30, and t=72 hours) demonstrates that Aβ has random coil structure at the beginning of aggregation (t=0 hours), then transitions to α-sheet structure at the end of the lag phase (t=30 hours). Finally, at the end of aggregation (t=72 hours), Aβ has β-sheet structure. D) Circular dichroism spectroscopy of three pre-incubated CsgA samples (t=1, t=32, and t=150 hours) demonstrates that CsgA has random coil structure at the beginning of aggregation (t=1 hour), then transitions to α-sheet structure at the end of the lag phase (t=32 hours). Finally, at the end of aggregation (t=150 hours), CsgA has β-sheet structure. From Bleem et al[43]. E) ThT signal of Aβ overlayed with toxicity demonstrates that toxicity peaks during the late lag phase of aggregation when α-sheet oligomers are present, then dissipates during the exponential and plateau phases. From Shea et al[3]. F) CD spectra of Aβ shows that an oligomeric sample taken from the late lag phase of aggregation produces similar spectra to that of a *de novo* α-sheet peptide, whose structure was confirmed by NMR to be α-sheet. From Shea et al[3].
